# Evolutionary game analysis of online game studios and online game companies participating in the virtual economy of online games

**DOI:** 10.1371/journal.pone.0296374

**Published:** 2024-01-24

**Authors:** Gong Zhang, Shulei Bi

**Affiliations:** 1 Research Center for Economy of Upper Reaches of the Yangtse River, Chongqing Technology and Business University, Chongqing, China; 2 Doctoral School of Entrepreneurship and Business, Budapest Business University, Budapest, Hungary; Zhejiang Gongshang University, CHINA

## Abstract

In the context of the new economic development in the post-pandemic era, "play" labor as an important component of digital work has become an inexhaustible driving force for the growth of the digital economy. Previous research has shown that "play" labor, as an emerging business model, can effectively promote the growth of the digital economy. However, there is a relative lack of research on the dynamic evolutionary game between "play" labor suppliers represented by game studios and online gaming companies. In this study, we applied the theoretical approach of dynamic evolutionary game theory to establish a game model depicting the evolution of both parties involved in the virtual economy of online gaming. The aim was to investigate the strategic selection mechanisms and influencing factors for game studios and online gaming companies participating in the virtual economy of online gaming. By analyzing the evolutionary game path, equilibrium points, and factors influencing the evolutionary game outcome, as well as conducting numerical simulation analysis using Matlab software, we found that the incremental gains and costs resulting from the strategic choices of online gaming companies and game studios in engaging in the virtual economy of online gaming affect the evolutionary outcomes. In addition, for the probability ratio of online game studios and online game companies choosing to participate in the virtual economy of online games, whether it is online game studios or online game companies, the larger the initialization ratio, the more likely the evolution result is to develop in a mutually beneficial direction. After an in-depth analysis and discussion of the evolutionary game results, relevant policy recommendations were proposed. We hope to provide a reference for promoting online game companies to strengthen the adequate supervision of online game studios’ participation in the virtual economy of online games and optimize and improve the virtual economic environment.

## 1. Introduction

Since the outbreak of COVID-19 in 2020, due to the government’s requirements for the prevention and control of the epidemic, people have had to start living and leisure ways such as home office and family entertainment [1, 2]. During COVID-19, although the physical industry as a whole was facing depression and recession, at the same time, online shopping, online education, live broadcast, online games, and other sectors had explosive growth [3–5]. As of the end of 2021, the user base of online gaming in China has reached 546 million, accounting for over 39% of the total population. Moreover, the total revenue generated by the Chinese online gaming market has exceeded 400 billion yuan. The continuous growth of the online game market has brought tremendous business opportunities to online gaming companies and third-party online gaming studios that rely on online games and player profits [6]. Third-party studios are commonly found in major online games, formed by a group of personnel with a large amount of gaming experience, resources, and technology, with a profit-oriented group [7]. Its scale varies, with small studios requiring only a few people and around ten computers to operate. The larger ones are recruited by specialized companies, with a population of 50–100 people and over 300 computers [8, 9]. These studios can survive in the gaming market thanks to an essential factor: the demand for game players. As the digital economy continues to expand, emerging industries based on the value creation model of "play" labor are gradually growing. For example, in the popular online role-playing game "World of Warcraft," some players wish to quickly acquire high-level equipment and complete multiplayer quests, but they may be limited by time and the availability of playing partners. To meet this demand, third-party online gaming studios provide services for players to quickly obtain equipment and complete quests. This has led to the rise of virtual transactions where players pay for gear and services. Third-party studios operate through platforms such as Taobao, Tmall, and WeChat, allowing players to directly purchase the desired products and exchange in-game currency, power leveling, and item trading. Additionally, similar to popular e-commerce websites like eBay and Amazon, most platforms implement a user rating system, enabling easier evaluation of the reputation and work quality of online gaming studios based on customer ratings and reviews on various websites [10].

Based on Daniel Kahneman and Amos Tversky’s "Prospect Theory," in the decision-making process, there may be a tendency for potential loss expectations (negative effects) to outweigh potential gain expectations (positive effects) [11]. Therefore, from the perspective of online gaming companies, although their attitude towards combating third-party online gaming studios is firm, if the direction and intensity of the crackdown are not well-managed, it is highly likely that unintended consequences will arise. However, this "prospect effect" primarily relies on decision-making processes under normal circumstances and does not specifically consider the dynamics of individual differences, cognitive biases (which may be influenced by context and time), as well as predictions and guidance for the future of the research subjects. A significant body of behavioral economics theories indicates that economic participants generate different mechanisms of competition and cooperation at various stages of development [12–14]. In particular, when an economic agent has multiple identities, they can engage in multiple games, requiring the use of Nash equilibrium theory to analyze the optimal strategies and possible outcomes of each game [15–17]. Although China’s online gaming industry has maintained rapid growth in recent years, it also faces fierce competition. Due to market saturation and increasing competitors, certain online gaming companies may encounter challenges of slowing revenue growth, particularly in specific game categories or market segments. As market demand increases, the number of online gaming studios in China has also increased, displaying an emerging virtual economic development model characterized by "play" labor. However, the lack of timely and effective industry regulation by government departments in tandem with virtual economic development has hindered the maximization of economic benefits for both parties involved. There is also a phenomenon of "bad money driving out good money." It is evident that different stages of virtual economic development have different behavioral effects and exert different influences on the parties participating in the virtual economy. Theoretically, one party in a game can weaken another party’s controllable resources by strengthening its competitive advantage, resulting in a self-serving outcome [18]. Alternatively, they can achieve a "win-win" outcome through cooperative linkage [19]. Therefore, how online gaming studios and companies can achieve their development expectations through competition and cooperation in different stages of development requires new theoretical and empirical research, and it also becomes a new perspective for studying the factors and mechanisms influencing the development of "play" labor within the context of digital economic development.

Therefore, this study focuses on the following questions: How do online gaming companies and online gaming studios interact in different stages of virtual economic development? What are the influencing factors that affect their specific strategic choices? How can the optimal strategy be selected to achieve the respective expectations of both parties? In light of these questions, this paper starts from the perspective of dynamic evolutionary game theory and examines the interactive relationship between online gaming companies and online gaming studios. It analyzes the specific factors that influence both parties in the dynamic game process and explores the optimal solutions for strategic choices in the game between online gaming companies and studios. By answering these questions, our aim is to reveal the impact mechanisms of game theory on the virtual economy and provide theoretical basis and practical guidance for policy formulation and development in related industries.

This paper’s potential contribution lies in the following aspects: (1) it provides a new perspective from the level of dynamic evolutionary game theory to enrich the practical research on "play" labor; (2) it offers a quantitative analysis approach with case studies focusing on the specific characteristics of "play" labor; (3) it presents a new perspective for government and regulatory authorities to have a comprehensive understanding of productive gaming practices and regulation in the context of virtual online gaming worlds.

## 2. Literature review

### 2.1 Digital labor and "Play" labor

The earliest research on digital labor can be traced back to the field of Western communication political economy, with Dallas Smythe’s theory of the audience commodity as the starting point for studying digital labor [20]. For a long time, audience commodities were not extensively studied in the production domain but rather confined to the cultural sphere. Ana C. et al. (2013) studied audience labor from an economic perspective, suggesting that culture, including leisure, entertainment, and culture involving information flow and data processing, is ultimately part of the material production domain determined by material history [21]. Subsequently, scholars gradually shifted their focus on the production domain when studying audience labor, recognizing the production process of these commodities as a form of labor [22]. Building upon Dallas Smythe’s theory of the audience commodity, research on digital labor expanded from the field of cultural media to intangible product domains such as information, knowledge, and emotions, and further extended to various production domains related to digital technologies. With the increasing application and maturity of technologies such as automation, artificial intelligence, the Internet of Things, and cloud computing, the role of digital technology in the production of intangible products has become increasingly significant, drawing attention from scholars. Terms such as "consumptive labor [23]," "productive labor [24]," and "prosumption of the Internet and social media [25] " have been proposed. Although these terms differ from the concept of digital labor, they all refer to the same phenomenon where information products are created due to the collection of information during entertainment or consumption, thus implying the presence of labor characteristics.

Currently, digital labor and "play" labor remain hot topics in current research, with relevant literature offering in-depth perspectives and research results on these two forms of labor. Firstly, digital labor is defined as activities that involve online platforms and virtual communities through the Internet and digital technologies. In contrast, "play" labor emphasizes meaningful participation and creativity in gamified environments [26]. The rise of these two forms of labor in the digital age has brought about a series of impacts and challenges. On the one hand, the boundaries between digital labor and "play" labor have become increasingly blurred as more and more people turn entertainment, gaming, and social activities into a source of income, further expanding the scope of these two forms [27]. On the other hand, blurring boundaries have also brought about certain issues, such as the safeguarding of labor rights, working conditions, and income uncertainty. Additionally, digital labor and "play" labor have had wide-ranging effects on society and individuals [28–30]. At the societal level, they have profound implications for employment structures, work patterns, and labor ecosystems [31]. However, these two forms of labor also give rise to a series of social issues, such as safeguarding labor rights, economic inequality, and the relationship between humans and technology [32]. At the individual level, participants in digital labor and "play" labor seek value and meaning in terms of self-realization, social identity, creative development, and entertainment experiences [33]. However, people’s perceptions and pursuits of these forms of labor may vary due to individual differences [34]. In summary, while digital labor encompasses "play" labor in its scope and essence, they differ significantly in specific manifestations, external influences, and meanings. Previous research perspectives have focused more on qualitative studies of the concepts, blurred boundaries, societal impacts, and individual value identification of digital labor and "play" labor, neglecting the exploration of specific case studies and quantitative influences of these labor forms.

### 2.2 Online game companies and game studios

Online game companies and game studios are two important entities in the gaming industry, and existing literature provides research findings on their development, collaboration, operation, and talent management. Firstly, previous research has found differences in positioning and development paths between online game companies and game studios [35–37]. Online game companies, specialized in the development and operation of online games, typically have strong financial and resource support [38]. On the other hand, game studios focus on creativity and independent development, often smaller in scale but more flexible [39]. Secondly, regarding collaboration models and innovation between online game companies and game studios, Yoo et al. (2012) proposes that they can achieve complementary advantages and enhance creative and development capabilities through various forms of collaboration such as independent development, outsourcing, and intellectual property sharing [40]. Damien and Denis (2017) discovers that online game companies involve game studios after the release of a new game, providing trial play feedback for game operation and effectively promoting game publicity and marketing by leveraging the influence of game studios, thus forming a synergistic effect with players [41]. Furthermore, innovative collaboration models and game design are crucial for achieving common development goals [42]. Additionally, Wei et al. (2023) points out the differences in operation and profit models between online game companies and game studios. Online game companies mainly rely on the release and operation of large-scale online games to generate revenue, while game studios obtain profits through game sales, IP licensing, and customized development [43]. However, due to the differences in industry status, when game studios choose to ignore the "game rules" set by online game companies for their own interests, they may become targets for precautionary measures and retaliation by online game companies [44]. It is evident that existing research mainly focuses on the differences and influences between online game companies and studios, with a focus on their operational models, internal structures, and qualitative dimensions of mutual influence, lacking in-depth research from a quantitative perspective on the dynamic evolutionary game between the two and their extended impacts.

### 2.3 The application of evolutionary game theory in the digital economy

Currently, evolutionary game theory has been widely applied in various industries [45, 46], such as enterprise behavior [47–49] and environmental domains [50, 51]. Its application in the field of digital economy is an important research topic. Existing literature has reviewed the relationship between evolutionary game theory and the digital economy, providing in-depth analysis and insights [52, 53]. Firstly, researchers have found limitations of traditional economic theory in understanding behavior and change in the digital economy, while evolutionary game theory offers a better perspective and framework [54]. Evolutionary game theory can simulate the evolution process of various strategies in the digital economy, revealing behavior patterns such as cooperation, competition, and coordination among participants [55]. For example, Tang, et al. (2023) constructed a trilateral evolutionary game model to analyze the influencing factors among manufacturing firms, governments, and digital technology platforms [56]. Wang, et al. (2023) explored the collaborative strategies and evolutionary patterns of value co-creators in the digital service ecosystem of the construction industry, achieving efficient collaborative value co-creation [57]. Secondly, researchers have also discussed issues of game strategies and profit allocation in the digital economy [58]. Participants in the digital economy face various choices of game strategies, such as cooperation [59], competition [60], merger [61], and so on. The application of game theory can analyze the impact of different strategies on individual and overall benefits and study how to distribute benefits fairly, promoting the sustainable development of the digital economy [62]. He et al. (2022) defined stakeholders in digital content innovation activities and analyzed their behavioral logic and influencing factors to explore the impact mechanism of diverse participating entities on the process of digital content innovation [63]. In addition, existing literature has focused on exploring innovation, technological development, and policy design in the digital economy [64, 65]. Evolutionary game theory can facilitate the understanding of technological choices and evolutionary paths in the process of digital economy innovation, promoting the acceleration and optimization of the innovation process [66]. Moreover, by understanding the game behavior and strategic choices among participants, flexible and adaptive policy measures can be formulated for the digital economy, promoting market competition, encouraging innovation, and improving overall efficiency [67]. Therefore, it can be observed that the application of evolutionary game theory in the digital economy primarily focuses on the relationship and interaction between the two, with a research perspective that is more inclined towards understanding the impact mechanisms among governments, businesses, and consumers.

As the digital economy continues to evolve, "play" labor has gradually become an important factor in digital work. Additionally, "play" labor has also transformed the business operation models within the gaming industry. However, despite the attention given in the literature to the value production and income patterns changes brought by "play" labor in the digital economy era, there are still several shortcomings. Firstly, there is a lack of clarity regarding the specific boundaries and impacts of "play" labor. The existing research mainly focuses on how individuals can obtain variable income and rewards through audience labor, neglecting the interactions and influences of new individual economic sectors (such as game studios and other spontaneous organizations or units) in the digital economy. Secondly, while attention has been given to the commercial differences and win-win collaborations, as well as profit motives, between online gaming companies and game studios, there is a lack of a comprehensive quantitative analysis on the game of game companies and game studios from a dynamic evolutionary perspective throughout the entire process. Thirdly, although the significance of evolutionary game theory in studying the relationships within the digital economy has been recognized, there is a lack of in-depth analysis from the perspective of online gaming companies and game studios. In reality, emerging private economic units adopting "play" labor as a business model are gradually increasing. These units can be further classified into various modes based on specific entertainment content, such as game leveling, gaming companions, live streaming, and promotion. Moreover, not only in the gaming industry but other industries related to mass entertainment are also experiencing explosive growth, such as tourism live streaming and digital task distribution. Therefore, it is worth conducting in-depth research and analysis on the multiple interactions and influence mechanisms within the virtual economy using "play" labor as a background.

## 3. Analysis of game evolution and stability strategies between online game studios and online game companies

In terms of strategic choices in the game between online gaming studios and companies, we can take "League of Legends" as an example. Riot Games is the development company behind this popular multiplayer online game. In the game, players can improve their rankings and skill levels through competitive modes. However, there are some online gaming studios or individuals offering a service called "Elo Boosting" aimed at helping players improve their rankings. These Elo Boosters usually use skilled players to play on the players’ accounts to boost their rankings. This behavior is considered a violation of the game rules and terms of service by Riot Games. Riot Games has taken various measures to combat Elo Boosting. They have strengthened detection and anti-cheating mechanisms within the game, monitoring and banning accounts involved in Elo Boosting. Additionally, they collaborate with the community to raise awareness through promotional and educational activities, advising players not to use boosting services in order to maintain the fairness and competitive environment of the game. However, the results have been limited.

Furthermore, Electronic Arts’ "FIFA" series of football games also faces similar issues. There are online gaming studios or individuals offering virtual currency trading services, commonly known as "Coin Selling," aimed at helping players obtain more virtual currency (such as in-game coins) to purchase players and improve their team’s strength. These Coin Sellers collect a large amount of virtual currency and then sell it to players in exchange for real money. Electronic Arts takes a strict stance against Coin Selling activities, viewing them as violations of the game rules and terms of service. Although they have implemented several measures to combat Coin Sellers, they have not entirely eliminated the "wild growth" of online gaming studios. Similar situations repeatedly occur in various online games, such as Valve’s "Counter-Strike: Global Offensive" and Epic Games’ "Fortnite."

On one hand, if online gaming companies attempt to sanction online gaming studios through legal means, it is usually difficult for law enforcement to intervene unless the studios are directly harming the company’s computer systems through actions like scripting or cheating. Legal action initiated by gaming companies may result in lighter convictions, and if they were to sue every online gaming studio, it would require high litigation costs and only provide temporary solutions. On the other hand, online gaming studios do not operate openly like non-player characters (NPCs) in massively multiplayer online games. In other words, while the operations of gaming companies are transparent, studios often operate in the shadows. Many game development companies, in their attempts to crack down on studios, end up inadvertently targeting regular players as well. Sometimes, the negative effects of combating studios are more apparent. Excessive crackdowns can significantly decrease the popularity of an online game, especially affecting the interest of "whale" players who spend a significant amount of real money on in-game purchases. This can result in many players quitting the game, something that gaming companies do not want to see. Therefore, it is challenging for gaming companies to excessively regulate online gaming studios and they often only issue warnings for minor infractions. Excessive regulation can affect player interest, while lack of regulation can lead to negative reviews. This is the dilemma faced by many gaming companies today.

Similarly, online gaming studios that engage in the virtual economy of online games still face significant risks. Firstly, excessive exploitation of virtual in-game currency can cause inflation in the game’s economy, leading to a substantial devaluation of the currency. This can result in the studios being unable to recover their initial investment costs from virtual currency trading, particularly in a non-fully transparent market where the studios cannot accurately predict player demand for virtual currency. As a result, they often struggle with issues of supply and demand. Secondly, if gaming companies implement exceptionally strict measures, online gaming studios may face the risk of having their reserve accounts permanently banned, resulting in significant sunk costs and operational risks. Thirdly, due to the lack of robust regulations in the legal framework governing virtual currency transactions in online games, if the virtual currency reserves of online gaming studios are illegally stolen by hackers or other individuals/organizations, it becomes challenging for the studios to seek compensation effectively. Additionally, their trading models lack legal protection, putting them at a disadvantage if players present evidence and litigate on trading platforms. Therefore, online gaming studios face corresponding dilemmas when deciding whether or not to participate in the virtual economy of online games.

This article mainly considers bounded rationality from the perspective of evolutionary game theory, takes group preference behavior as the research object, and combines game theory methods with dynamic evolution [68–70]. Based on our field research and industry analysis of Chinese online gaming studios and gaming companies, we have found that although the development and promotion of online gaming studios’ participation in the virtual economy may vary across different regions, the main actors and objectives are generally the same. The primary goal is to obtain extremely rare and valuable virtual items or in-game currency in online games and then convert them into real currency through transactions. Since game players are their main source of real currency transactions, the impact of gaming companies on the operational efficiency and gold output models of online gaming studios is more evident and necessary. At the same time, considering the assumptions regarding the probability of strategy selection in evolutionary game theory, it is clear that the behavior and decision-making processes of both online gaming studios and gaming companies are influenced by various factors. These factors include the potential risks, rewards, and regulations associated with participating in the virtual economy, as well as the strategies adopted by their counterparts. Maximizing profit and minimizing risks are key considerations for both parties [71]. Therefore, we selected the virtual economy model of online games with the participation of game players with absolute demand as the research object and proposed the following assumptions for the model:

①. Gaming party: Assuming that one party in the game is an online gaming company; The other side of the game is the online gaming studio

②. Strategy: The strategy of online game companies is whether to actively supervise online game studios to participate in the virtual economy of online games, with a strategy set of [supervision, no supervision]; The strategy of the online game studio is whether to participate in the virtual economy of online games, and the strategy set is [participate, not participate].

③. Income matrix: Assuming that the proportion of online gaming companies choosing an active regulatory model is y (0<y<1). When online gaming companies decide to participate in the dynamic regulatory model, the profits obtained by online gaming companies can be expressed as E1; Due to the implementation of an active regulatory model, the operating environment of online games has been purified, resulting in an enhanced gaming experience for players, which will lead to more players registering game accounts and generating incremental income R; Implementing an active regulatory model requires a significant investment in manpower and equipment costs C1 (such as increasing the number of online GMs to ensure timely and effective handling of player feedback in the game); Due to excessive active regulation, ordinary players are mistakenly mistaken for online game studios being wrongly banned, resulting in an additional cost of excessive regulation of C2; The legal cost of prosecuting some online gaming studios for illegal activities is C3; When online gaming companies do not choose to start an active regulatory model, the revenue of online gaming companies is E2; The relationship between online gaming companies and online gaming studios is a simple one between players and game manufacturers. Due to the active regulatory model implemented by online gaming companies, the number of online gaming studios in the game is much lower than that of game players, which is precisely why online gaming studios can achieve favorable results. The number of game accounts banned by online gaming companies due to active regulation of online gaming studios is far smaller than the number of new game players. Therefore, E1 is more significant than E2. From a long-term perspective, the total added benefits will outweigh the cost incurred, i.e., E1-E2+R>C1+C2+C3.

AsThe evolution of selection strategies between online gaming suming that the proportion of online gaming studios that choose to participate in the virtual economy of online gaming is x (0<x<1). The income obtained by online game studios after participating in the supply of virtual economy for online games is expressed as P1; As the number of online game players increases, the incremental income generated by online game studios participating in the virtual economy of online games is K; The technology learning cost invested by the online gaming studio to fully earn virtual game coins is S1; The cost of staffing and equipment invested is S2; The infringement cost incurred in the face of potential judicial lawsuits filed by online gaming companies is S3; The profit of online gaming studios not participating in the virtual economy of online games is P2. Taking World of Warcraft as an example, online gaming studios that do not participate in the virtual economy of online games can receive equivalent real currency rewards by providing technical services such as character training and upgrading, completing tasks and achievements, and playing on behalf of arenas to other players. This specialized service is relatively private and involves privacy issues of personal account security for game players; Online gaming companies are usually unable to take adequate measures to regulate games; When online gaming studios are involved in the virtual economy of online gaming, in addition to providing technical services to generate income, they can participate in various ways such as hoarding game materials to buy low and sell high, collecting game coins to invest more, or short selling operations in maximizing their interests. Furthermore, it can be seen that P1>P2. From a long-term perspective, the total added benefits far outweigh the cost incurred, i.e., P1-P2+K>S1+S2+S3, and the specific benefit matrix Table 1 can be obtained.

**Table 1:** Evolutionary game benefit matrix.

Game Player		Online gaming companies	
		Active supervision (y)	Not actively regulated (1-y)
Online Game Studio	Join(x)	E1+R-C1-C2-C3	E2
		P1+K-S1-S2-S3	P2+K-S1-S2
	Not-Join (1-x)	E2+R-C1-C2	E2
		P2	P2

The effectiveness of online game studios participating in the virtual economy of online games is:

$$\begin{array}{c} U_{11}=y(P_{1}+K-S_{1}-S_{2}-S_{3})+(1-y)(P_{2}+K-S1-S2)\\ =y(P_{1}-P_{2}-S_{3})+P_{2}+K-S_{1}-S_{2} \end{array}$$ (1)

The utility of online game studios not participating in the virtual economy of online games is:

$$U_{12}=yP_{2}+(1-y)P_{2}=P_{2}$$ (2)

The average effect of online gaming studios is:

$$U_{1}=xU_{11}+(1-x)U_{12}=x[y(P_{1}-P_{2}-S_{3})-S_{1}-S_{2}]+P_{2}$$ (3)

The replication dynamic equation of the game player in the online game studio is:

$$F(x)=\frac{dx}{dt}=x(U_{11}-U_{1})=x(x-1)[S_{1}+S_{2}-K-y(P_{1}-P_{2}-S_{3})]$$ (4)

Similarly, it can be seen that the effectiveness of active regulatory strategies adopted by online gaming companies is:

$$\begin{array}{c} U_{21}=x(E_{1}+R-C_{1}-C_{2}-C_{3})+(1-x)(E_{2}+R-C1-C2)\\ =x(E_{1}-E_{2}-C_{3})+E_{2}+R-C_{1}-C_{2} \end{array}$$ (5)

The effectiveness of adopting an inactive regulatory strategy by online gaming companies is:

$$U_{22}=xE_{2}+(1-x)E_{2}=E_{2}$$ (6)

The average utility of online gaming companies is:

$$\begin{array}{l} U_{2}=yU_{21}+(1-y)U_{22}\\ \;=y[(x-1)(C_{1}+C_{2}-E_{2}-R)-x(C_{1}+C_{2}+C_{3}-E_{1}-R)]-E_{2}(y-1) \end{array}$$ (7)

The replication dynamic equation of the online game company’s game player is:

$$F(y)=\frac{dy}{dt}=y(U_{21}-U_{2})=y(y-1)[C_{1}+C_{2}-R-x(E_{1}-E_{2}-C_{3})]$$ (8)

The evolution of selection strategies between online gaming studios and companies can be systematically described using Eqs (4) and (8) above. The Jacobian matrix of the system is:

$$J=(\begin{array}{cc} \left(x‐1)[S_{1}+S_{2}‐K+y(P_{2}‐P_{1}+S_{3})]+x[S_{1}+S_{2}‐K+y(P_{2}‐P_{1}+S_{3})\right] & x(x‐1)(P_{2}‐P_{1}+S_{3})\\ y(y‐1)(C_{3}‐E_{1}+E_{2}) & y[C_{1}+C_{2}‐R+x(C_{3}‐E_{1}+E_{2})]+(y‐1)[C_{1}+C_{2}‐R+x(C_{3}‐E_{1}+E_{2})] \end{array})$$ (9)

When F (x) = 0 and F (y) = 0 to obtain five dynamic equilibrium points: O (0,0); A (0,1); B (1,0); C (1,1); D (m,n); Where m = - (C1+C2-R)/(C3-E1+E2), n = - (S1+S2-K)/(P2-P1+S3). According to the assumption, point D should be located within the first quadrant of the coordinate axis.

## 4. Evolutionary game model analysis of online game studios and online game companies participating in the virtual economy of online games

Dynamic analysis of replication in online game studios: Based on the obtained equilibrium points O, A, B, and C, they form the boundary {(x, y) | x = 0,1; y = 0,1 |} of the evolutionary game domain. Therefore, the besieged area OABC is the equilibrium solution domain of the game between both parties, that is, OABC = {(x, y) | 0 ≤ x ≤ 1, 0 ≤ y ≤ 1}, and there is also an equilibrium point D in this region that satisfies the condition. Due to the non-asymptotic stability of D in the dynamic replication system composed of online game studios and online game companies, it is necessary to discuss the asymptotic stability of O, A, B, and C. Obviously, each equilibrium point in the system corresponds to an evolutionary game equilibrium. After substituting the balance points into the Jacobian matrix, calculate their eigenvalues to obtain the corresponding Jacobian matrix eigenvalues of the equilibrium points, as shown in Table 2.

**Table 2:** Eigenvalues of Jacobi matrix.

Equilibrium	Characteristic value 1	Characteristic value 2
O (0,0)	K-S1-S2	R-C2-C1
A (0,1)	C1+C2-R	K+P1-P2-S1-S2-S3
B (1,0)	S1+S2-K	E1-C2-C3-C1-E2+R
C (1,1)	P2-P1-K+S1+S2+S3	C1+C2+C3-E1+E2-R
D (m,n)	F	-F


$$F=\frac{{\left[(S_{1}+S_{2}-K)(P_{2}-P_{1}+S_{3})(C_{3}-E_{1}+E_{2})(C_{1}+C_{2}-R)(P_{2}-P_{1}-K+S_{1}+S_{2}+S_{3})(C_{1}+C_{2}+C_{3}-E_{1}+E_{2}-R)\right]}^{\left(1/2\right)}}{\left(C_{3}*P_{2}-C_{3}*P_{1}+E_{1}*P_{1}-E_{1}*P_{2}-E_{2}*P_{1}+E_{2}*P_{2}+C_{3}*S_{3}-E_{1}*S_{3}+E_{2}*S_{3}\right)}$$

Further analysis of m and n can yield the following four scenarios:

① When 0 < m < 1, 0 < n < 1, i.e., E1-E2+R > C1+C2+C3, P1-P2+K > S1+S2+S3, the total revenue increased by the online gaming company after implementing an active regulatory strategy exceeds the cost. At the same time, the online gaming studio also participates in the online gaming virtual economy strategy, and the increased revenue exceeds the cost spent. According to the results in Table 2, the local stability analysis results of the game system can be further organized, as shown in Table 3. The results in Table 3 show that the system has two equilibrium points, namely the O point and the C point, indicating that the evolution result is Reporting analysis results. Either the online game studio or the online game company choose to participate in the strategy of the online game virtual economy at the same time, or they both do not choose the strategy at the same time.

**Table 3:** Local stability analysis of game system.

Equilibrium	Tr(J)	Det(J)	Local stability
O(0,0)	-	+	ESS
A(0,1)	+	+	Unstability
B(1,0)	+	+	Unstability
C(1,1)	-	+	ESS
D(m,n)	0	-	Saddle Point

Note: Tr (J) and Det (J) represent the trace and determinant of Jacobi Matrix, respectively. Same below

② When 0 < m < 1, n > 1, i.e., E1-E2+R > C1+C2+C3, P1-P2+K < S1+S2+S3, the total revenue increased by the online game company after choosing an active regulatory strategy exceeds the cost, while the income of the online game studio after deciding to participate in the online game virtual economy strategy cannot repay all the costs. At this point, the equilibrium point is point O (0,0), and the result of the evolutionary game is that neither online gaming companies nor online gaming studios will choose to participate in the virtual economy strategy of online gaming.

③ When m > 1, 0 < n < 1, i.e., E1-E2+R < C1+C2+C3, P1-P2+K > S1+S2+S3, the total increase in revenue after the online gaming company chooses to regulate the strategy actively is less than the cost, while the income after the online gaming studio decides to participate in the online gaming virtual economy strategy exceeds the total cost. The result of the evolutionary game is that neither online gaming companies nor online gaming studios will participate in the virtual economy strategy of online gaming, as its equilibrium point is still point O (0,0).

④ When m>1 and n>1, i.e., E1-E2+R < C1+C2+C3, P1-P2+K < S1+S2+S3, neither online gaming company nor online gaming studio can benefit from the added profits after implementing the selection strategy. The evolutionary game results in both parties giving up their choices.

The specific analysis results of the above four situations are shown in Table 4:

**Table 4:** Stability analysis results under different conditions.

Equilibrium		①		②		③			④	
O (0,0)	Tr(J)	-	ESS	-	ESS	-	ESS	-		ESS
	Det(J)	+		+		+		+		
A (0,1)	Tr(J)	+	Unstability	△	Saddle Point	+	Unstability	△		Saddle Point
	Det(J)	+		-		+		-		
B (1,0)	Tr(J)	+	Unstability	+	Unstability	△	Saddle Point	△		Saddle Point
	Det(J)	+		+		-		-		
C (1,1)	Tr(J)	-	ESS	△	Saddle Point	△	Saddle Point	+		Unstability
	Det(J)	+		-		-		+		
D (m,n)	Tr(J)	0	Saddle Point	0	Saddle Point	0	Saddle Point	0		Saddle Point
	Det(J)	-		+		+		-		

Note: Among them, △ indicates that the symbol cannot be determined

**Fig 1:**
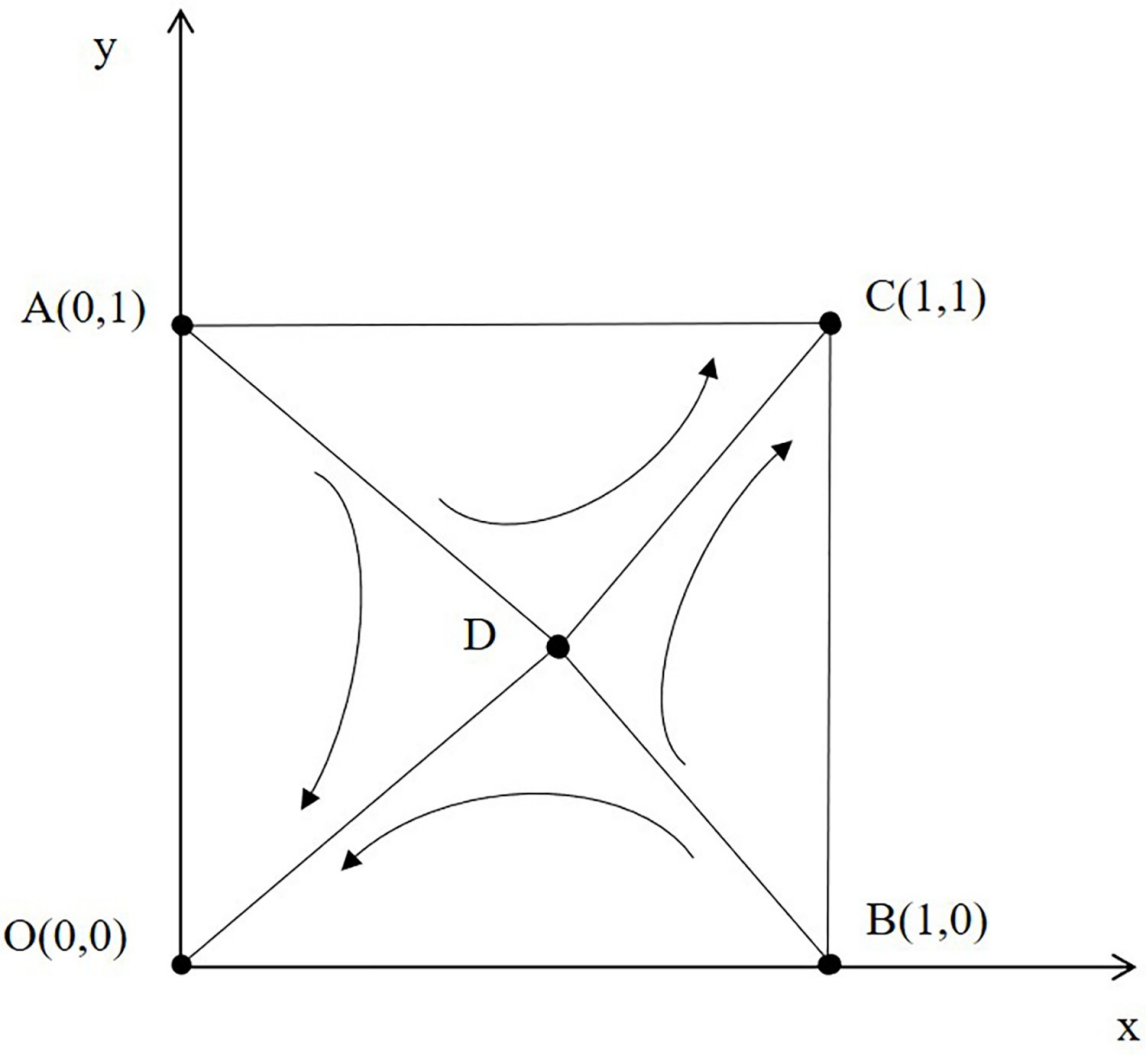
Evolution phase diagram of online game studio and online game company.


Combining the analysis results of Tables 2–4, it can be found that the evolution results in the first scenario (when 0<m<1, 0<n<1) are uncertain, and analyzing the first scenario has significant practical and theoretical significance. The phase diagram for the first scenario can be drawn based on the previous stability analysis:

Fig 1 shows that the quadrilateral AOBC can be divided into two parts: quadrilateral ACBD and quadrilateral ADBO. If the initial state is in ACBD, the evolution results tend to develop towards point C (1,1), which means that in this case, online gaming companies will choose active regulatory strategies, and online gaming studios will also choose to participate in the virtual economy strategy of online gaming actively. If the initial state is in ADBO, the evolution results will develop towards point O (0,0), so both online gaming companies and studios tend to refuse to choose any strategy. If the area of the quadrilateral ACBD increases, the chances of the initial state being in this region will also increase, leading to a higher probability of stable strategy C (1,1). The evolution path can be further explored by analyzing the area changes of quadrilateral ACBD, and the factors that affect the area can be used as factors that affect the evolution results.

$$S_{ACBD}=1-0.5m-0.5n=1-0.5[\frac{\left(C_{1}+C_{2}-R\right)}{\left(E_{1}-E_{2}-C_{3}\right)}+\frac{\left(S_{1}+S_{2}-K\right)}{\left(P_{1}-P_{2}-S_{3}\right)}]$$ (10)

According to Eq (10), the factors that affect the area of the quadrilateral ACBD are the factors that affect the evolution of virtual economic behavior in online games. Therefore, further analysis can be conducted:

If the revenue generated by online gaming companies increases by E1, the smaller the denominator of m, the larger the value, and thus the area of the quadrilateral ACBD will also increase. The larger the size, the higher the probability of the initial state being located within that area, and the results of the evolutionary game are more inclined to develop towards equilibrium point C (1,1). Therefore, it is concluded that both online gaming companies and online gaming studios will choose to participate in the online gaming virtual economy strategy.

If an online gaming company adopts an active regulatory strategy, the smaller the difference between the human equipment cost C1 and the high regulatory cost C2, and the incremental income R, the larger the area of the quadrilateral ACBD, which is a reverse change relationship. The result is also towards point C (1,1), similar to the above results.

If the litigation cost C3 of online gaming companies is smaller, the area of the quadrilateral ACBD will increase, and there is a reverse relationship between the two. The result also approaches point C (1,1), consistent with the above results.

Suppose the income P1 of online game studios participating in the virtual economy of online games increases. In that case, the denominator of n will increase, and then the value of n will decrease. Still, the area of the quadrilateral ACBD will increase, resulting in a reverse relationship between the two. The evolution result is also towards point C (1,1), and both parties in the game will choose to implement strategies.

If the difference between the learning costs S1 and human equipment costs S2 of online game studios and the incremental benefits K of participating in the virtual economy of online games is smaller, the area of the quadrilateral ACBD will be more prominent, and the result tends to develop towards equilibrium point C (1,1). Both parties then choose to implement strategies.

If the legal cost S3 of the online game studio is minor, the area of the quadrilateral ACBD will increase, and there is a reverse relationship between the two. The result is also towards point C (1,1), where both parties in the game choose to implement strategies.

## 5. Numerical simulation analysis of the evolutionary game between online game studios and online game companies participating in the virtual economy of online games

To more intuitively analyze the evolution trend of online gaming companies and online gaming studios in selecting strategies for participating in the virtual economy of online games, numerical simulation was conducted on the evolutionary game model of both parties using Matlab software, and the simulation results were analyzed when various parameters changed [72]. In order to set the initial parameter values of the model, we conducted extensive online searches on Baidu, explored internet forums, and interviews with industry professionals to select appropriate research subjects. Based on preliminary survey results, we decided to select online gaming studios with high ratings and positive customer reviews on platforms such as Taobao, 5173, and Pinduoduo as our research subjects. We chose online gaming studios as sampling units and selected studios with rankings higher than the average on the chosen websites as our initial contact points. The selection of online gaming studios also took into account their geographic locations to represent different types of cities/regions in China to the maximum extent possible.

We then obtained contact information, mainly phone numbers and WeChat IDs, and requested on-site visits. After explaining our research objectives, approximately 50% of the online gaming studios agreed to let us conduct on-site visits and agreed to be interviewed. In the end, six online gaming studios participated in our field research, with a total of 18 individual participants. The selected online gaming studios are distributed in five cities of different scales across the country—Shanghai (large), Beijing (large), Guangzhou (medium), Chongqing (small).

As for the selection of online gaming companies, we targeted the network gaming companies with the largest proportion of business operations as identified from our selection of online gaming studios. Based on our preliminary research on online gaming studios, we found that the majority of their income came from games such as "Fantasy Westward Journey," "World of Warcraft," "Nishuihan," and "Dungeon Fighter Online." Correspondingly, the network gaming companies were NetEase (Hangzhou) Limited and Tencent Technology (Shenzhen) Limited (although some online games are developed by foreign companies, current regulations in China require domestic companies to operate as agents). As large internet-listed companies, most of their data is publicly available. Therefore, by combining the publicly released financial data from these two companies, interview records with the online gaming studios, and considering the ideal state based on parameters set in evolutionary game theory, we have established the parameter settings for this study.

The initial probability, x = 0.5, y = 0.5, is primarily based on references [73, 74]. The values of parameters E1 = 600, E2 = 300, C1 = 30, C2 = 40, C3 = 50, R = 80, K = 80, S1 = 100, S2 = 50, S3 = 50, P1 = 300, and P2 = 150 are derived from research interviews and publicly available data.

**Fig 2:**
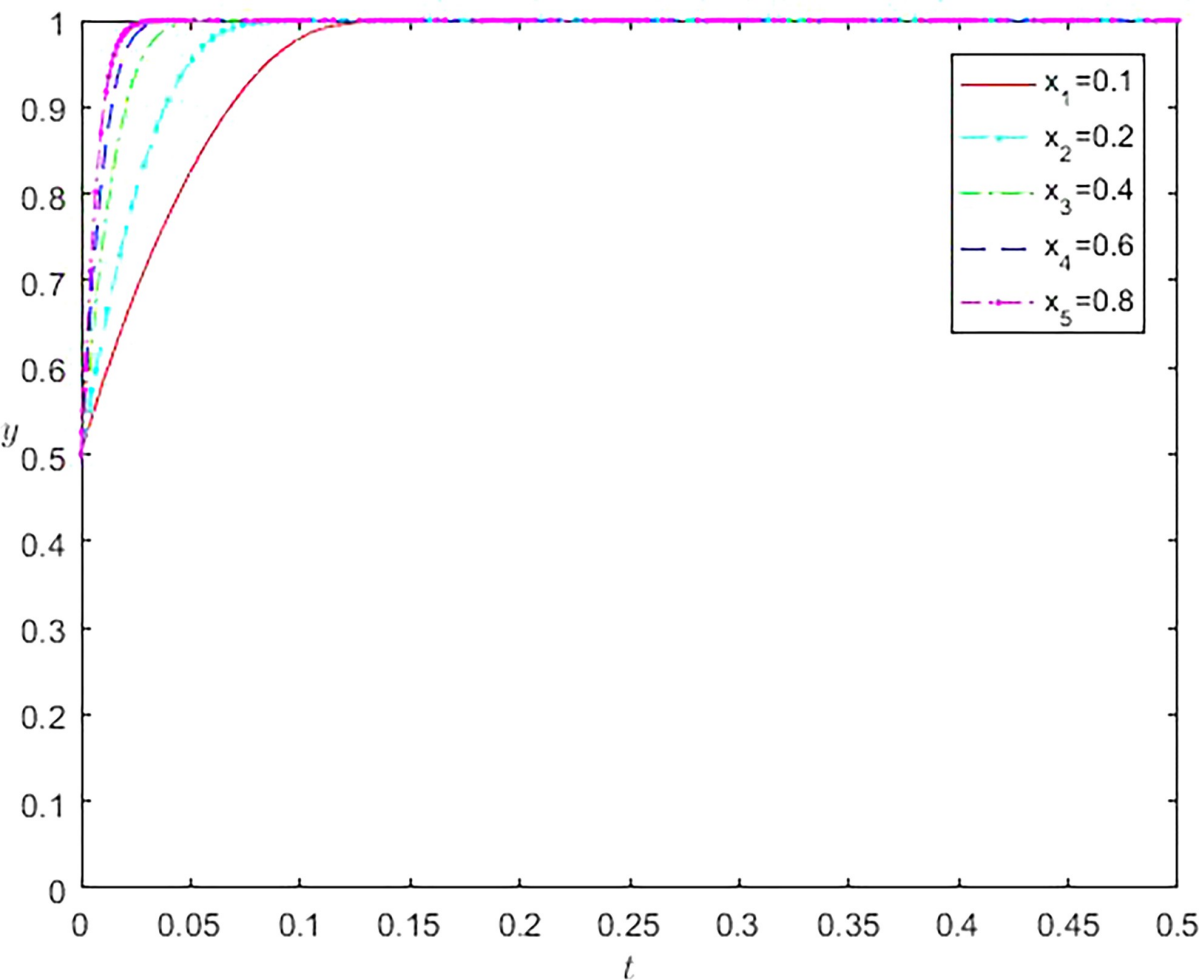
The impact of different initial proportions of online gaming studios on the evolution of online gaming company selection behavior.

**Fig 3:**
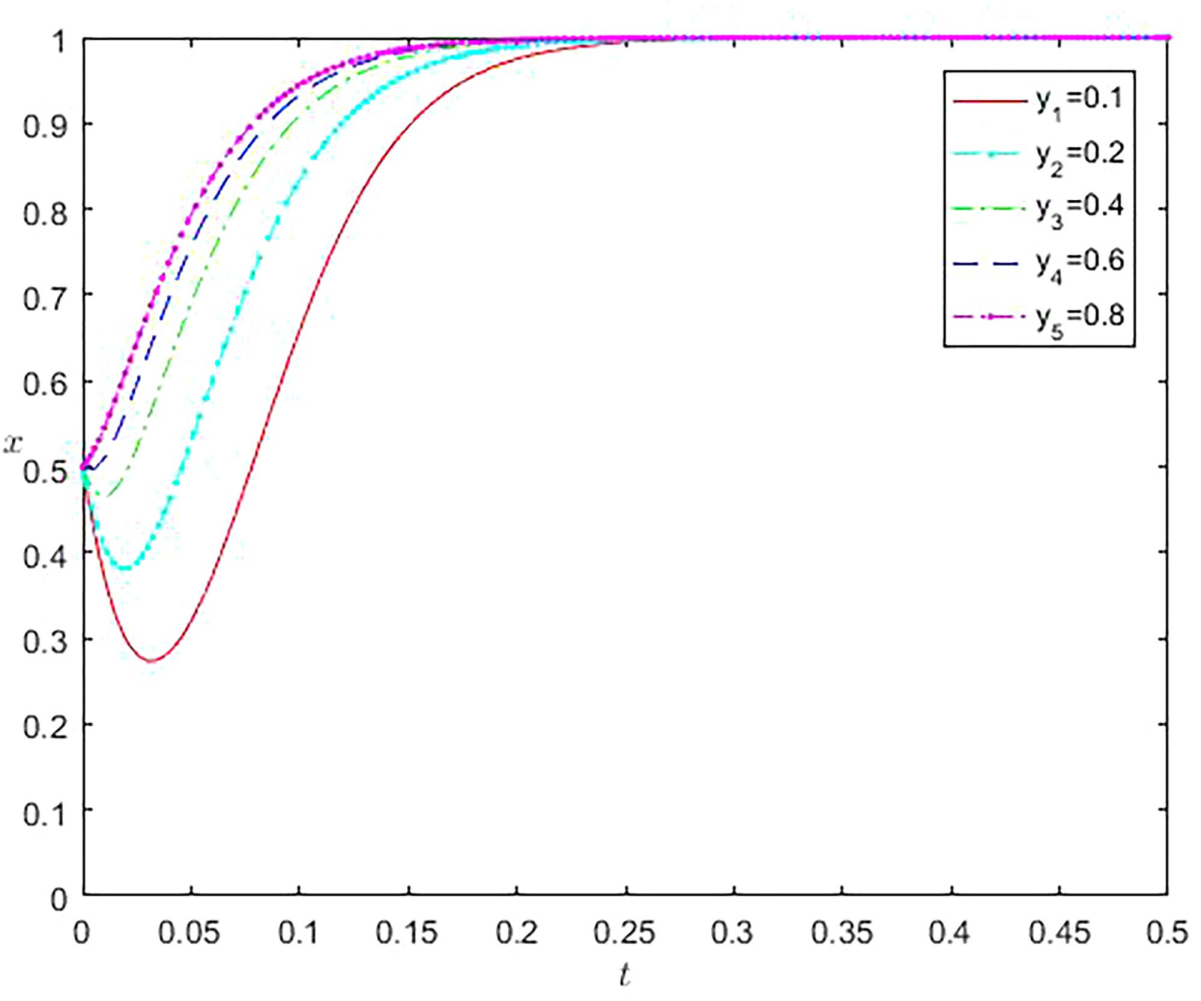
The impact of different initial proportions of online gaming companies on the evolution of online gaming studio selection behavior.

As the initial population proportion varies, the evolution results of dynamic games also vary. The different initial ratios affect the time it takes for the game to reach equilibrium, and the time it takes to get a stable state is shorter when the initial proportion is close to the equilibrium point compared to when the balance is not close to the equilibrium point. The result is shown in Fig 2. By changing the initial proportion x value of online gaming studios that choose to participate in the virtual economy strategy of online gaming, it can be found that the dynamic evolution results of online gaming companies have also undergone significant changes. As the proportion x increases, the time to reach a stable state continues to shorten. Similarly, when constantly changing the initial balance y value of online gaming companies, it can be observed that as the y value continues to increase, the time to reach a stable state also continuously shortens, as shown in Fig 3.

**Fig 4:**
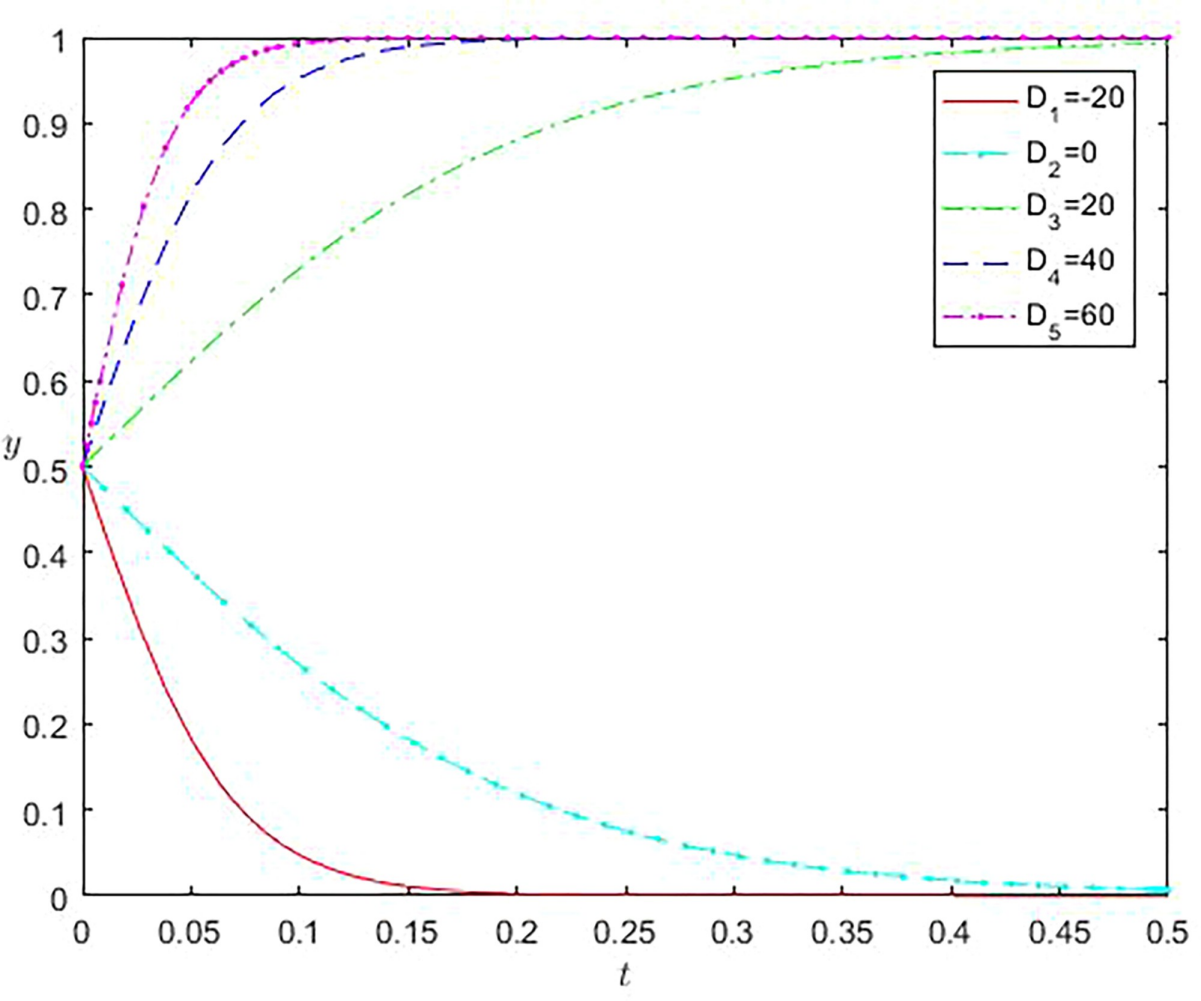
Impact of incremental revenue offsetting incremental cost difference of online game studio on evolution results.

A critical influencing factor considering whether to choose the implementation strategy is the incremental income R and K after implementing the strategy. Assessing the total costs of both parties after implementing the strategy and the conclusion of Eq (10). We use D to represent the degree of offset between the cost of online gaming studios and incremental costs, i.e., D = K-S1-S2. A more significant value of D indicates a tremendous difference between total and incremental costs. Fig 4 shows that when the D values are -20 and 0, the y-value results are zero. As the D value continues to increase, online gaming studios gradually switch strategies and choose strategies to participate in the virtual economy of online gaming. At the same time, the slope under different values shows that the speed of selecting such strategies is constantly accelerating.

**Fig 5:**
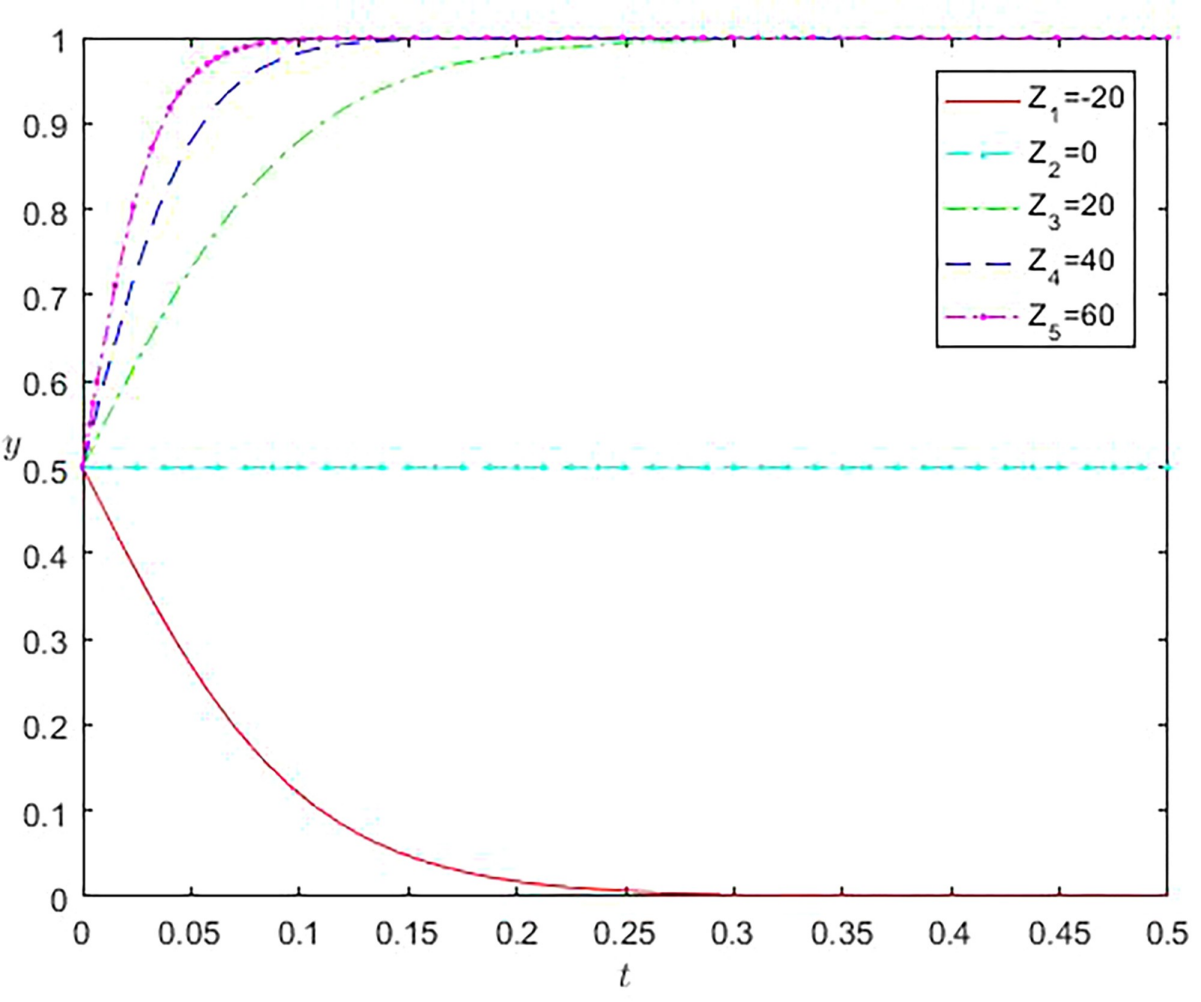
Impact of incremental revenue offsetting incremental cost differences of online game companies on evolution results.

Similarly, when an online gaming company chooses an active regulatory strategy, Z represents the difference between incremental benefits and incremental costs under the strategy selected by the company, i.e., Z = R-C1-C2. The larger the Z value, the more significant the difference between total benefits and incremental costs. From Fig 5, it can be observed that when the Z-value is -20, the Y-value tends to be zero. As the Z-value continues to increase, online gaming companies also begin to adjust their strategies and choose to adopt active regulatory behavior. At the same time, the larger the Z-value, the larger the slope, indicating that the adjustment time for online gaming companies to adopt active regulatory strategies will gradually shorten.

## 6. Discussion

On the one hand, the mode of online game studios participating in the online game virtual economy strategy is theoretically in line with the new labor economy participation mechanism with "play" as the process under the digital economy. However, there are many ways to realize this economic participation mechanism. One is that high-level players represented by technology participate in International competitions or projects and receive prize money in return [75], And display high-level game technology in the live room to get gifts and rewards [30]; Second, it is necessary to test the completeness of the game before it is launched, which is also a kind of digital labor. It is essential to determine whether the game itself has unreasonable logic and whether there are game vulnerabilities. Such a test also has the characteristics of playing labor [76]; Third, represented by the gold coin farmer, he paid to fight for others or got a piece of equipment to exchange, the lowest level of digital labor. However, this form of digital delivery is the most extensive and visible, which is also one of the subjects of this paper. Based on the work they are completing, we can now see that some things can be redefined as value in the digital environment. This value is not purely a value of productive labor defined by Marx at that time [77–79]. Today, we may have to redefine the "value," not to say that Marx’s original things are obsolete, and the productive labor is still there. However, in the digital space, it will form a new kind of labor that cannot be seen in our original framework, and this kind of labor needs to be redefined [80, 81].

With the Internet economy’s continuous development, the virtual industry’s new economic sector is gradually growing, and the online game virtual economy is only a part of this industrial model. First, the virtual economy of online games has investment value and growth potential in theory and practice and can bring real economic benefits to participants. Secondly, the virtual economy of online games has strong liquidity and transaction convenience, which can provide strong support for developing the digital economy. In addition, the online game virtual gold coin can also promote the innovation of virtual finance and the development of virtual industry and provide new ideas and power for developing the digital economy [82]. But the virtual economy of online games also faces many challenges. First of all, due to the lack of supervision and risk control of the online game virtual economy, market chaos and speculation are prone to occur under the manipulation of bad online game studios. Secondly, the volatility of the online game virtual economy is significant, and the price is easily affected by market sentiment and information, which is easy to lead to the loss of participants. In addition, the virtual economy of online games also has problems, such as technical and network security risks, and effective measures need to be taken to control risks [83].

According to previous research, the dynamic evolutionary game process between online game companies and online game studios tends to choose the direction of strategy development simultaneously, which is consistent with the situation in reality. When the online game company first launched an online game, the number of game players was small, and the online game company would not implement stringent regulatory measures. At the beginning of online games, the online game studio did not have a deep understanding of the development mechanism of online game virtual economy, so it would only choose to provide some technical services and take advantage of the hardware or software advantages relative to ordinary game players to obtain economic benefits. With the game’s increasing operation time and popularity, the number of players who began to enter online games has been growing. Based on the understanding of game technology, the online game studio has already started to have a more professional knowledge of the development mode of online game virtual economy. To obtain more realistic benefits, online game studios began participating in all aspects of the online game virtual economy. However, the deepening of online game studios’ participation in the online game virtual economy harmed the online game environment, resulting in the decline of players’ sense of game experience, and they began to complain and continuously give feedback to game companies. Online game companies began to choose to implement positive regulatory strategies to respond to the opinions of game players; This is consistent with the results of evolutionary games in this paper. Although active regulation can limit the probability of online game studios’ participation in online game virtual economy strategy to a certain extent, if excessive dynamic regulation occurs, it may affect the interests of ordinary and krypton gold players and the economic interests of online game companies.

To fundamentally solve the control of online game studios over the online game virtual economy, it may be necessary to change the source and the regulation of the online game virtual economy. Companies usually do not interfere with the game’s virtual economy in most online games. Online game companies can directly solve the immediate needs of players in the game in some ways. In that case, it will effectively alleviate the impact of online game studios on the online game virtual economy from another dimension [84, 85]. For example, taking the world of Warcraft as an example, after many online game studios entered the game, the prices soared due to the studios’ massive excavation of game gold coins, and the game’s virtual economy system collapsed. By actively setting up the market trading mechanism of game points (real currency) and game virtual gold coins at the game auction house, the inflation of the virtual game economy was alleviated to a certain extent. However, the online game studio only partially disappeared but took many methods, such as price reduction and economies of scale, to fight back [86]. Therefore, online game companies still need to innovate regulatory processes and take effective regulatory measures to protect players’ interests. In essence, the online game studio is also part of the player group, but it is different from other ordinary players because of the particularity of its game motivation. Therefore, it should appropriately carry out online game virtual economic activities to comply with laws and game operation regulations. Excessive mining of game gold coins will also accelerate the early termination of the life cycle of online games. At the same time, the government or industry associations should also modify and improve the relevant laws and regulations or industry regulations in this gray field, adjust the legal and economic relationship between online game companies and online game studios, restrict illegal gains, and protect legal digital labor achievements.

## 7. Conclusion

This paper mainly studies the evolution of the choice behavior of online game companies and online game studios under the premise of bounded rationality, considers the influencing factors of the choice behavior of online game companies and online game studios to participate in online game virtual economy strategy, constructs the income matrix and dynamic replication equation when they choose to participate in online game virtual economy strategy, analyzes the evolution path of the behavior of both sides under different strategies, and uses MATLAB software to carry out the numerical simulation. Simulation results have shown the following trends: (1) As the marginal benefits of participating strategies increase, there is a growing tendency to engage in online gaming virtual economy strategies; (2) The implementation of active regulation by gaming companies, with higher costs in terms of manpower and equipment, as well as excessive regulatory costs, and the higher learning costs and manpower and equipment costs for online gaming studios in participating in the virtual economy, will hinder the selection of strategies for both parties to engage in the online gaming virtual economy; (3) For the probability ratio of online gaming studios and gaming companies choosing to participate in the virtual economy of online gaming, both parties are more likely to develop in a direction that benefits both sides when the initial ratio is larger.

The implications of our research should be considered within the limitations of the study. Although we have made efforts to overcome these limitations, there are still some unavoidable constraints. The first limitation is the selection of game entities. This study only analyzed the game companies and online gaming studios from a two-player game perspective. However, it is evident that game players and governments, as third or fourth parties, have significant influence on both game companies and online gaming studios. In the future, a more in-depth analysis can be conducted from the perspective of three-party or four-party games. Secondly, the parameter selection in this study focused mainly on research conducted within China. It would be interesting to include relevant cases from other developed countries as parameter references, which may yield more insightful conclusions. Lastly, this research only analyzed the strategic choices of game companies and online gaming studios from the perspective of evolutionary game theory. If more continuous panel data samples can be obtained in the future, a microeconometric analysis can be conducted to empirically test the specific motivations behind the evolutionary game strategy selection and delve into the mechanisms influencing these choices.

Based on the research findings of this study, we propose the following policy recommendations:

Considering that online gaming studios are emerging players in the digital economy era, the evolutionary game process between the two sides drives them to choose strategies that are more advantageous to themselves. Therefore, it is necessary for the government or industry associations to strengthen regulation of online gaming studios and game companies as neutral third parties. On one hand, strict regulations and policies should be formulated to prevent online gaming studios from engaging in illegal activities such as cheating and fraudulent transactions, thus safeguarding fairness and healthy development of the gaming industry. At the same time, the operations of game companies should be regulated to ensure compliance with tax regulations, paying taxes according to the corresponding rates and provisions, in order to maintain the healthy development and fair competition of the gaming industry. On the other hand, supervision over game companies should be strengthened, encouraging them to establish sustainable and legal virtual economy models. They should provide players with virtual goods and services through legitimate channels, thereby reducing the existence of illicit transactions. Additionally, according to the simulation results, the higher the investment costs for both parties, the more it hinders them from choosing strategies to participate in the online gaming virtual economy. Therefore, both game companies and online gaming studios should strengthen internal management, implement measures for cost control, establish standardized operating systems based on modern business models, and eliminate illegal and irregular behaviors, while actively exploring new sources of revenue growth. In summary, it is important for the government, game companies, and online gaming studios to collaborate actively, strengthen regulation, promote legal operation, and safeguard the interests of the gaming industry and players through various measures such as education and technological means, ensuring the sustainable and healthy development of the gaming industry.
